# Surgical outcome of percutaneous transhepatic gallbladder drainage in acute cholecystitis: Ten years’ experience at a tertiary care centre

**DOI:** 10.1007/s00464-021-08573-0

**Published:** 2021-08-20

**Authors:** Szabolcs Ábrahám, Illés Tóth, Ria Benkő, Mária Matuz, Gabriella Kovács, Zita Morvay, András Nagy, Aurél Ottlakán, László Czakó, Zoltán Szepes, Dániel Váczi, András Négyessy, Attila Paszt, Zsolt Simonka, András Petri, György Lázár

**Affiliations:** 1 Department of Surgery, University of Szeged, Szeged, Hungary; 2 Department of Clinical Pharmacy, University of Szeged, Szeged, Hungary; 3 Central Pharmacy and Emergency Care Department, University of Szeged, Szeged, Hungary; 4 Central Pharmacy Department, University of Szeged, Szeged, Hungary; 5 Radiology Department, University of Szeged, Szeged, Hungary; 6 First Department of Internal Medicine, University of Szeged, Szeged, Hungary; 7 Department of Surgery, University of Szeged, Albert Szent-Györgyi Health Centre, Semmelweis u. 8., 6725 Szeged, Hungary

**Keywords:** Acute cholecystitis, Laparoscopic cholecystectomy, Mortality, Conversion rate, Percutaneous cholecystostomy

## Abstract

**Background:**

Percutaneous transhepatic gallbladder drainage (PTGBD) plays an important role in the treatment of elderly patients and/or patients in poor health with acute cholecystitis (AC). The primary aim of this study is to determine how these factors influence the clinical outcome of PTGBD. Moreover, we assessed the timing and results of subsequent cholecystectomies.

**Patients and Methods:**

We retrospectively examined the results of 162 patients undergoing PTGBD between 2010 and 2020 (male–female ratio: 51.23% *vs.* 48.77%; mean age: 71.43 ± 13.22 years). Patient’s performance status and intervention outcomes were assessed with clinical success rates (CSR) and in-hospital mortality. The conversion rate (CR) of possible urgent or delayed, elective laparoscopic cholecystectomies (LC) after PTGBD were analysed.

**Results:**

PTGBD was the definitive treatment in 42.18% of patients, while it was a bridging therapy prior to cholecystectomy (CCY) for the other patients. CSR was 87.97%, it was only 64.29% in grade III AC. In 9.87% of the cases, urgent LC was necessary after PTGBD, and its conversion rate was approximately equal to that of elective LC (18.18 *vs.* 17.46%, respectively, *p* = 0.2217). Overall, the post-PTGBD in-hospital mortality was 11.72%, while the same figure was 0% for grade I AC, 7.41% for grade II and 40.91% for grade III. Based on logistic regression analyses, in-hospital mortality (OR 6.07; CI 1.79–20.56), clinical progression (OR 7.62; CI 2.64–22.05) and the need for emergency CCY (OR 14.75; CI 3.07–70.81) were mostly determined by AC severity grade.

**Conclusion:**

PTGBD is an easy-to-perform intervention with promising clinical success rates in the treatment of acute cholecystitis. After PTGBD, the level of gallbladder inflammation played a decisive role in the course of AC. In a severe, grade III inflammation, we have to consider low CSR and high mortality.

Percutaneous transhepatic gallbladder drainage (PTGBD) is an invasive radiological method that has been used from the early 1980s [1–5], and it is now an essential part of treatment for acute cholecystitis (AC). Ultrasound (US)-guided PTGBD is a technically easy-to-perform method with a relatively high clinical success rate. According to the literature, the technical success rate of PTGBD is approximately 95% during AC treatment, whereas the clinical success rate ranges between 56% and 100% [6–10].

Nowadays, the Tokyo Guidelines 2018 recommendations form the standard in treating AC [11]. Compared to the Tokyo Guidelines 2013 (TG13) [12], the 2018 (TG18) version further clarifies the role of PTGBD in treating AC. In grade II, moderate AC urgent/early laparoscopic cholecystectomy (LC) is the primary therapy of choice. If the patient is unsuitable for surgery due to his general condition and/or does not respond adequately to antibiotics and general supportive care, PTGBD becomes necessary. According to the Tokyo Guidelines 2018, the indication for PTGBD in grade III AC is determined by the patient’s performance status (Charlson comorbidity index (CCI) [13], the American Society of Anesthesiologists (ASA) physical status [14]) based on negative predictive factors (jaundice and neurological and respiratory dysfunction) and favourable organ system failure (FOSF; cardiovascular and renal organ failure).

Complicated AC is a relatively high-mortality disease [15]. Emergency or early LC is a surgery with great technical difficulties, and it is accompanied by a high conversion rate [16, 17], a chance of biliary duct injury and high mortality [8]. The aim of PTGBD is to provide an alternative treatment option to high-risk patients with moderate (grade II) or severe (grade III) AC. In some cases, when the patient is not even fit for elective surgery, PTGBD can provide a definitive therapeutic solution. However, most of the time, it serves as a bridging therapy before elective LC.

Managing AC is a multidisciplinary task which aims to treat the disease most effectively by avoiding septic complications. In many cases, despite the detailed recommendations in the guidelines, it is difficult to decide which treatment strategy is the most ideal for the patient. Nevertheless, whatever treatment or path we choose, the objective is to avoid complications and decrease mortality and thus to increase survival.

The success of AC treatment may be influenced by several factors, such as general condition, comorbidities, patient age and level of gallbladder inflammation. The aim of this study is to ascertain how these factors influence the success of PTGBD, the timing of subsequent cholecystectomies and their outcomes. In addition, it is necessary to further clarify the role and place of PTGBD in the complex treatment algorithm of AC.

## Materials and methods

Ethical permission (81/2020-SZTE) for the study was obtained from the Regional Human Biomedical Research Ethics Committee of the University of Szeged.

We retrospectively examined abdominal ultrasound (US)-guided PTGBD interventions performed with AC indication at the University of Szeged for a ten-year period from 2010 to 2020. Patients who underwent percutaneous transhepatic gallbladder aspiration or endosonography-guided gallbladder drainage or computer tomography (CT)-guided PTGBD were excluded from the study. We did these exclusions to provide a homogenous study population in terms of the used interventional radiology method (i.e. only ultrasound-guided PTGBD). Moreover, nine patients who had a history of hepato-pancreatic-biliary malignancy prior to PTGBD or who were diagnosed with it after the procedure as well as patients who received further treatment after PTGBD outside the University of Szeged were excluded. After exclusions, data were analysed from 162 patients with PTGBD.

In radiologically confirmed AC patients, the TG13 and TG18 recommendations were followed when indicating PTGBD [18, 12, 11].

The severity of inflammation was determined retrospectively based on the TG18/TG13 severity grading for acute cholecystitis defined in the Tokyo Guidelines 2018 [19]. The severity of AC-related inflammation in each patient was classified as grade I (mild), II (moderate) and III (severe). Based on abdominal ultrasound, the indications for PTGBD were grouped as follows: acute acalculous cholecystitis (AAC), acute calculous cholecystitis (ACC), empyema vesicae felleae (EVF), hydrops vesicae felleae (HVF) and covered perforated cholecystitis (PC) [20].

Sex and age group (18–65 years or over 65 years) distribution and patient’s performance status were determined: the ASA score (I–VI) was determined for each patient, and patients were classified into three groups based on the Charlson comorbidity index (CCI) as follows: CCI 0, CCI 1–3 and over CCI 4. Based on the time elapsed between the onset of complaints and PTGBD, patients were grouped into three categories (0–72 h, three days to one week and beyond one week).

The average duration of drain presence after PTGBD was assessed. The need for endoscopic retrograde cholangiopancreatography (ERCP) during hospitalization and after hospital discharge time over an average were followed for a five-year period. The indications of ERCP (non-decreasing biliary excretion, sepsis (including cholangiosepsis), biliary obstruction (BO)) and its results were assessed. The need for urgent CCY due to the rapidly deteriorating clinical condition of the patients after PTGBD was determined.

Three endpoints were determined in terms of clinical and surgical outcomes of PTGBD. The clinical success rate (CSR) of PTGBD (number of clinically regressive cases after PTGBD X 100/[total number of PTGBD procedures – number of technically unsuccessful procedures]) was calculated. Clinical regression was determined by remission of patient’s symptoms, improvement in inflammatory markers (leukocyte count, CRP and PCT) and radiological (US or abdominal CT) regression. As a routine practice, we followed up the patients with control abdominal ultrasound after the PTGBD everyday/every second day or rarely with CT. We check the position of the inserted tube/drain and the possible regression or progression of the gall bladder inflammation (thickness of the gallbladder wall, pericholecystic fluid, etc.).

CSR was assessed according to different patient sexes, age groups, TG18/13 AC severity grades, CCI and time elapsed between the onset of complaints and hospital admission were analysed.

In addition to CSR, the technical success rate (TSR) of PTGBD (technically successful procedure × 100/total procedures) was also calculated. We interpreted invasive radiological interventions where we observed drain failure (occlusion, drain displacement, improper tube positioning, etc.) as technically unsuccessful PTGBD.

As a second endpoint in terms of clinical outcome, we analysed the proportion of CCYs after PTGBD and the need for possible emergency surgeries. We examined the proportion of PTGBD reported as final therapy (no need for CCY) and as a bridging therapy (i.e. the percentage of elective CCY performed in patients who responded well to drainage). All elective CCY surgeries performed after hospital discharge during an average five-year follow-up period were analysed. In terms of surgical outcome, we determined the proportion of primary open cholecystectomy, laparoscopic cholecystectomy (LC) and conversion after LC during both emergency and elective CCY surgery. Based on the above, the conversion rate (CR) of LCs (number of converted LCs × 100/[number of total surgeries – number of primary open cholecystectomies] and the laparoscopic success rate (LSR) (number of LCs/numbers of total surgeries) were calculated. Elective surgeries were further divided into two groups according to the time elapsed between PTGBD and the CCY surgery (performed between three to six weeks and after six weeks). In these groups, the previous parameters (CR and LSR) were also determined. Possible bile duct injury during CCY was examined as well.

Finally, as a third endpoint in terms of clinical and surgical outcome, we calculated the in-hospital mortality and procedure mortality (directly related to PTGBD, such as bleeding, embolism and other organ injury). We further analysed in-hospital mortality in relation to different patient or intervention characteristics.

### Statistical analysis

Detailed descriptive statistics for continuous and categorical variables were reported. Welch’s t-test, one-way ANOVA, Pearson’s chi-squared test or Fischer’s exact test were used for the univariate analysis, as appropriate. We tested the association between negative patient outcomes (in-hospital mortality, clinical progression and emergency cholecystectomy) and patient’s performance status or ACC severity with a univariate method followed by logistic regression. Statistical analysis was performed using R 3.5.1.

## Results

Among the 162 patients who underwent PTGBD within the ten-year investigation period, there were nearly equal proportions of men and women (51.23% *vs.* 48.77%). Their mean age was 71.43 ± 13.22 years, and the majority of them (71.60%) was over 65 years of age. It should be noted that the age of patients who died after PTGBD during in-hospital time was significantly higher compared to the survival group (76.82 ± 9.77 *vs*. 71.16 ± 12.98 years). Mean age was significantly higher in more severe inflammation (grade I: 63.14 ± 16.52 years; grade II: 70.79 ± 13.14 years; grade III: 78.89 ± 7.22 years) and in patients who required emergency CCY than those who had elective CCY (74.75 ± 13.13 *vs.* 68.00 ± 11.05 years). In cases where no surgical procedure was performed, PTGBD served as definitive therapy. Mean age of these patients was 73.39 ± 15.39 years.

In addition to the high mean age, the majority of the PTGBD patients had a CCI above 4 (65.38%). The distribution of the AC severity grade was the following: grade I: 8.8%; grade II: 73.6%; and grade III: 17.6. Most frequently, PTGBD was called for due to abdominal US-confirmed ACC in 33.95% of the cases, covered cholecystitis perforation in 27.16% and AAC in 5.56% (Table 1). Hospital admission occurred between 72 h and one week after the onset of complaints in almost half of the cases (45.6%). In general, PTGBD was performed within 72 h in 39.71% of the cases, and beyond one week in 14.71%. TSR for PTGBD was 97.53%, procedure mortality was 0%, and CSR was 87.97%. The drain inserted was removed 11.65 ± 7.57 days after PTGBD on average. After PTGBD, 62 (42.18%) did not undergo subsequent CCY; drainage therefore proved to be a definitive therapy. 69 patients (46.94) had CCY, and 16 patients (10.88%) had emergency surgery due to the deteriorating clinical condition and progression. The mean timing of elective surgeries was 13.57 ± 10.89 weeks after PTGBD (Table 1).

**Table 1:** General patient and intervention characteristics

		*N*	%	Mean ± SD	Min–Max
*Age* (years)	30–65	46	28.4		
	65 +	116	71.6		
	Total	162	100	71.43 ± 13.22	33–95
*Sex*	Female	79	48.77		
	Male	83	51.23		
	Total	162	100		
*ASA score*	1	16	10.13		
	2	65	41.14		
	3	54	34.18		
	4	23	14.56		
	*NA*	4			
*CCI*	CCI 0	8	5.13		
	CCI 1–3	46	29.49		
	CCI 4 +	102	65.38		
	Total	156	100	4.21 ± 2.25	0–10
	*NA*	6			
*Time frame* (between onset of complaints and hospital admission)	0–72 h	54	39.71		
	72 h–1 week	62	45.59		
	Over 1 week	20	14.71		
	*NA*	26			
*Indication of PTGBD based on abdominal US* *N* = 140; 100%	AAC	9	5.56		
	ACC	55	33.95		
	EVF	17	10.49		
	HVF	37	22.84		
	PC	44	27.16		
*AC severity grade* (TG18/TG13)	I	14	8.81		
	II	117	73.58		
	III	28	17.61		
	*NA*	3			
*PTGBD TSR* *N* = 162 100%			97.53		
*PTGBD CSR* *N* = 162 100%			87.97		
Time of drain removal after PTGBD (days)		88		11.65 ± 7.57	1–42
	*NA*	*76*			
*Mortality after PTGBD*	Procedure mortality	0	0		
	In-hospital mortality	17	11.72		
*ERCP after PTGBD*	During hospital stay	21	13.46		
	After hospital discharge	4	2.56		
	There was no ERCP	131	83.97		
	*NA*	6			
*CCY after PTGBD*	Emergency CCY	16	10.88		
	Elective CCY	69	46.94		
	There was no surgery	62	42.18		
*BDI during CCY after PTGBD*		1	1.17		
*Time interval between PTGBD and CCY*	Emergency (days)	16	19.05	5.50 ± 12.56	0–52
	Elective (weeks)	68	80.95	13.57 ± 10.89	2–67
	Total	84	100	11.24 ± 10.92	0–67
	*NA*	*1*			

*AC* Acute cholecystitis, *AAC* Acute acalculous cholecystitis, *ACC* Acute calculous cholecystitis, *BDI* Bile duct injury, *CCY* Cholecystectomy, *CSR* Clinical success rate, *ERCP* Endoscopic retrograde cholangiopancreatography, *EVF* Empyema vesicae felleae, *HVF* Hydrops vesicae felleae, *NA* No data, *PC* Covered perforated cholecyst, *TSR* Technical success rate, *TG13/18* Tokyo Guidelines 2013 and 2018, *US* Ultrasound

CSR of PTGBD deteriorated significantly in patients over 65 years and in parallel with the increasing severity of the inflammation (Table 2). While basically all patients under 65 years of age experienced clinical regression, CSR was only 83.62% in patients over 65 years. In grade I inflammation, we also had complete clinical success in all patients; however, CRS was 92.04 in grade II and only 64.29% in grade III. The clinical regression varied inversely with the ASA score and a similar tendency could be observed for CCI; CSR was 100% for CCI 0, 88.37% for CCI 1–3 and 86.96% for CCI 4 + . There was no significant difference in CSR in relation to time elapsed between the onset of complaints and hospital admission (see Table 3).

**Table 2:** Indications and timing of endoscopic retrograde cholangiopancreatography (ERCP) after percutaneous transhepatic gallbladder drainage (PTGBD)

	ERCP indication	ERCP outcome	*N*
During in-hospital stay *N* = 20; 83.33%	Non-decreasing biliary excretion *N* = 12; 50.00%	BO: CBDS	5
		BO: Juxtapapillary diverticulum	2
		BO: SOD	2
		BO: Sclerosis of Vater’s papilla	1
		Irregular pancreatic anatomy	1
		BO: Mirizzi syndrome	1
	Cholangiosepsis N = 4; 16.66%	BO: CBDS	2
		BO: Duodenal stenosis	1
		BO: Biliary stent obstruction	1
	Increased biliary obstruction enzymes N = 3; 12.50%	BO: Juxtapapillary diverticulum	2
		BO: CBDS	1
	Sepsis	Abdominal gallbladder perforation	1
After hospital discharge *N* = 4; 16.67%	Increased biliary obstruction enzymes	BO: CBDS	2
	Cholangiosepsis	BO: CBDS	1
	Non-decreasing biliary excretion	Intrahepatic minor BDI	1
Total			24
*NA*			1

*BDI* Bile duct injury, *BO* Biliary obstruction, *CBDS* Common bile duct stone, *NA* No data, *SOD* Sphincter of Oddi dysfunction

**Table 3:** Technical success rate and clinical outcomes of percutaneous transhepatic gallbladder drainage (PTGBD) according to patient characteristics

		Clinical progression after PTGBD	Clinical regression after PTGBD	Technically unsuccessful PTGBD	Total	TSR %	CSR %	p*
Total		19	139	4	162	97.53	87.97	
Age (years)	30–65	0	42	4	46		100	0.003926
	65 +	19	97	0	116		83.62	
Sex	Female	11	65	3	79		85.53	0.5053
	Male	8	74	1	83		90.24	
ASA score	1	0	14	2	16		100	–
	2	12	52	1	65		81.25	
	3	4	49	1	54		92.45	
	4	3	20	0	23		86.96	
	*NA*		4		4			
CCI	CCI = 0	0	7	1	8		100	0.6372
	CCI = 1–3	5	38	3	46		88.37	
	CCI = 4 +	14	88	0	102		86.27	
	*NA*		6		6			
Time frame (between onset of complaints and hospital admission)	0–72 h	8	46	0	54		85.19	0.8191
	72 h–1 week	7	52	3	62		88.14	
	Over 1 week	2	17	1	20		89.47	
	*NA*	2	24		26			
AC severity grade (TG18/TG13)	I	0	14	0	14		100	0.0009995
	II	9	104	4	117		92.04	
	III	10	18	0	28		64.29	
	*NA*		3		3			

*AC* Acute cholecystitis, *NA* No data (*Pearson’s chi-squared test)

After PTGBD, ERCP was necessary in 15.43% of the cases (25 cases) (Table 2). The most common indication for ERCP was in cases, where no reduction in bile flow through the inserted gallbladder drain was seen. Irrespective of the indication for ERCP, choledocholithiasis was confirmed in 40% of the cases (Table 2).

Comparing emergency and elective CCY surgeries after PTGBD in terms of LSR and CR (Table 4), the proportion of primary open cholecystectomies in elective surgeries was much lower (5/16 (7.24%) *vs.* 5/69 (31.25%)). The CR of elective LCs (17.46%) was similar to that of emergency LCs (18.18%).

**Table 4:** Characteristics of cholecystectomies (CCY) performed after percutaneous transhepatic gallbladder drainage (PTGBD)

			LC	Converted LC	Primary open CCY	NA	Total	LSR (%)	p* LSR%	CR (%)
Total			61	13	10	1	85	71.76	*–*	17.57
CCY after PTGBD	Emergency		9	2	5		16	56.25	0.1367	18.18
	Planned CCY		52	11	5	1	69	75.36		17.46
		within 3 to 6 weeks	5	1	1		8	62.50	0.3969	16.67
		after 6 weeks	47	10	4		61	77.05		17.54

*LSR* Laparoscopic success rate (number of LCs/total number of surgeries), *CR* Conversion rate (number of converted LCs × 100/[total number of surgeries – number of primary open cholecystectomies]) (*Fischer’s Exact Test)

If we further analyse elective and emergency CCYs (Table 5), it can be seen that emergency CCYs were mainly performed in older patients with higher CCI or more severe AC.

**Table 5:** The characteristics of emergency and elective cholecystectomies (CCY) performed after percutaneous transhepatic gallbladder drainage (PTGBD)

		Elective CCY after PTGBD	Emergency CCY after PTGBD	Total	*P*
		*N* = 69 (100%)	*N* = 16 (100%)		
Age (years)	30–65	27 (39.13%)	2 (12.5%)	29	*p* value = 0.0762 Fisher’s exact test
	65 +	42 (60.87%)	14 (87.5%)	56	
Sex	Female	29 (42.03%)	10 (62.5%)	39	*p* value = 0.1698 Fisher’s exact test
	Male	40 (57.97%)	6 (37.5%)	46	
ASA score	1	6 (8.7%)	2 (12.5%)	8	*p* value = 0.7576 Pearson’s chi-squared test with simulated *p* value (based on 2000 replicates)
	2	37 (53.62%)	10 (62.5%)	47	
	3	23 (33.33%)	3 (18.75%)	26	
	4	3 (4.35%)	1 (6.25%)	4	
CCI	CCI = 0	3 (4.41%)	1 (6.25%)	4	*p* value = 0.8236 Pearson’s chi-squared test with simulated *p* value (based on 2000 replicates)
	CCI = 1–3	29 (42.65%)	5 (31.25%)	34	
	CCI = 4 or 4 +	36 (52.94%)	10 (62.5%)	46	
	*NA*	1		1	
Time frame (between onset of complaints and hospital admission)	0–72 h	23 (36.51%)	6 (42.86%)	29	*p* value = 0.93 Pearson’s chi-squared test with simulated *p* value (based on 2000 replicates)
	72 h–1 week	30 (47.62%)	6 (42.86%)	36	
	Over 1 week	10 (15.87%)	2 (14.29%)	12	
	*NA*	6	2	8	
Indication of PTGBD based on abdominal US	AAC	2 (2.9%)	1 (6.25%)	3	*p* value = 0.7836 Pearson’s chi-squared test with simulated *p* value (based on 2000 replicates)
	ACC	25 (36.23%)	8 (50%)	33	
	EVF	5 (7.25%)	1 (6.25%)	6	
	HVF	16 (23.19%)	2 (12.5%)	18	
	PC	21 (30.43%)	4 (25%)	25	
AC severity grade (TG18/TG13)	I	9 (13.04%)	0 (0%)	9	*p* value = 0.0004998
	II	56 (81.16%)	9 (56.25%)	65	Pearson’s chi-squared test with simulated *p* value (based on 2000 replicates)
	III	4 (5.8%)	7 (43.75%)	11	

*AC* Acute cholecystitis, *AAC* Acute acalculous cholecystitis, *ACC* Acute calculous cholecystitis, *EVF* Empyema vesicae felleae, *HVF* Hydrops vesicae felleae, *NA* No data, *PC* Covered perforated cholecyst, *US* Ultrasound

In addition to the 0% procedure mortality directly associated with the PTGBD intervention, in-hospital mortality was 11.72% (Table 6). There was no significant difference in mortality between male and female patients; however, mortality showed a corresponding increase with the increasing score for both ASA score and CCI. The most prominent mortality was observed in AAC cases. In this scenario, five out of nine patients died with an in-hospital mortality of 55.56%, while mortality was only 6.00% for ACC. Mortality after elective surgery was 0%; however, if emergency CCY was required after PTGBD, we lost 14.29% of the patients.

**Table 6:** Survival and in-hospital mortality according to patient and intervention characteristics

Total		*N*		Survival		In-hospital mortality	NA	*p*
		162	128	(88.28%)	17	(11.72%)	17	–
Age (years)	30–65	46	36	(90.00%)	4	(10.00%)	6	0.7811
	65 +	116	92	(87.62%)	13	(12.38%)	11	
Sex	Female	79	58	(87.88%)	8	(12.12%)	13	1
	Male	83	70	(88.61%)	9	(11.39%)	4	
ASA score	1	16	15	(100.00%)	0	(0.00%)	1	sim *p* value = 0.001999
	2	65	55	(98.21%)	1	(1.79%)	9	
	3	54	42	(85.71%)	7	(14.29%)	5	
	4	23	15	(68.18%)	7	(31.82%)	1	
CCI	CCI = 0	8	6	(100.00%)	0	(0.00%)	2	sim *p* value = 0.02299
	CCI = 1–3	46	41	(100.00%)	0	(0.00%)	5	
	CCI = 4 +	102	79	(84.04%)	15	(15.96%)	8	
	*NA*	6						
Time frame (between onset of complaints and hospital admission)	0–72 h	54	46	(86.79%)	7	(13.21%)	1	sim *p* value = 0.1729
	3 days–1 week	62	52	(96.3%)	2	(3.70%)	3	
	Over 1 week	20	15	(88.24%)	2	(11.76%)	8	
	*NA*	26						
Indication of PTGBD based on abdominal US	AAC	9	4	(44.44%)	5	(55.56%)	0	—
	ACC	55	47	(94.00%)	3	(6.00%)	5	
	EVF	17	11	(91.67%)	1	(8.33%)	5	
	HVF	37	30	(88.24%)	4	(11.76%)	3	
	PC	44	36	(90.00%)	4	(10.00%)	4	
AC severity grade (TG18/TG13)	I	14	14	(100%)	0	(0%)	0	sim *p* value = 0.0004998
	II	117	100	(92.59%)	8	(7.41%)	9	
	III	28	13	(59.09%)	9	(40.91%)	6	
	*NA*	3						
CCY after PTGBD	Planned	69	66	(100%)	0	(0%)	3	0.0288
	Emergency	16	12	(85.71%)	2	(14.29%)	2	

*AAC* Acute acalculous cholecystitis, *ACC* Acute calculous cholecystitis, *EVF* Empyema vesicae felleae, *HVF* Hydrops vesicae felleae, *NA* No data, *PC* Covered perforated cholecyst, *PTGBD* Percutaneous transhepatic gallbladder drainage, *TG13/18* Tokyo Guidelines 2013 and 2018, *US* Ultrasound (*Fischer’s exact test and Pearson’s chi-squared test)

The logistic regression (Table 7) showed that the severity of AC inflammation had the highest odds for emergency CCY (OR 14.75; CI 3.07–70.81). The degree of inflammation also had a significant effect on clinical progression (OR 7.62; Cl 2.64–22.05) and on in-hospital mortality (OR 6.07; CI 1.79–20.56). CCI had a significant odds ratio only for in-hospital mortality (similarly to the results of the univariate analysis).

**Table 7:** Logistic regression between negative patient outcomes (in-hospital mortality, clinical progression and emergency cholecystectomy) and patient’s performance status or AC severity

		B	S.E	df	*P*	OR (95% CI)	Model characteristics
In-hospital mortality *N* = 141	CCI	0.562	0.168	1	0.001	1.75 (1.26–2.44)	Nagelkerke R-squared = 0.345; Correct predictions = 87.2%
	AC severity grade	1.803	0.623	1	0.004	6.07 (1.79–20.56)	
	Constant	−9.154	1.955	1	0.000		
Clinical progression after PTGBD *N* = 152	CCI	−0.001	0.136	1	0.995	1.00 (0.77–1.3)	Nagelkerke R-squared = 0.199; Correct predictions = 87.5%
	AC severity grade	2.031	0.542	1	0.000	7.62 (2.64–22.05)	
	Constant	−6.533	1.287	1	0.000		
Emergency CCY after PTGBD *N* = 84	CCI	−0.124	0.182	1	0.495	0.88 (0.62–1.26)	Nagelkerke R-squared = 0.273; Correct predictions = 84.5%
	AC severity grade	2.691	0.800	1	0.001	14.75 (3.07–70.81)	
	Constant	−6.812	1.611	1	0.000		

*AC* Acute cholecystitis, *B* Regression coefficient, *CCI* Charlson comorbidity index, *df* Degree of freedom, *OR* Odds ratio, *CI* Confidence interval

## Discussion

Therapy of AC is a complex multidisciplinary task. A number of factors must be considered to make a therapeutic decision. According to the Tokyo Guidelines 2018 recommendations, we should consider the patient’s age, general condition, comorbidities, the beginning of his or her complaints and the severity of the gallbladder inflammation [11].

US-guided PTGBD has been used for almost four decades and nowadays plays an important role in emergency care for AC [1, 3, 4, 2, 21]. PTGBD is a relatively easy procedure with a high technical success rate, used mainly in moderate-to-severe AC if the patient is not fit for surgery or does not respond adequately to antibiotics and general supportive care [8]. Although a patient’s advanced age is not an absolute contraindication for acute early CCY, it may still be a determinant of complex AC treatment success [22]. Our study showed that PTGBD was mainly performed in older patients (mean age: 71.43 ± 13.22 years) with AC and CSR, with 83.62% of the patients being over 65 years. Higher mean age was also observed in more severe inflammation. Consequently, it is likely that the less elderly age group is more likely to experience clinical regression, while CSR is literally 100% for patients under 65 years.

Based on our findings, grade II and grade III inflammation mainly occurred in older patients. Among a population with advanced age, a higher CCI may accompany a potentially increased mortality rate. In our study, in-hospital mortality was 15.96% in CCI 4 + patients and 0% in CCI 0–3. Based on previous reports, AC mortality varied with the severity of the inflammation. While 30-day mortality was 1.1% in mild inflammation (grade I), it was 0.8% in moderate (grade II) and 5.4% in severe inflammation [23]. In-hospital mortality after PTGBD was relatively high (11.72%) in our study. High mortality after PTGBD was confirmed by a systematic review conducted by Winbladh et al*.*, which showed 15.4% total mortality (30-day mortality or in-hospital death) [8]. Dimou et al*.* reported 24% in-hospital mortality after 563 PTGBDs [24]. Although the guidelines recommend PTGBD for elderly or critically ill patients as well as in grade II–III inflammation [22, 11], mortality of approximately 41% was observed in grade III inflammation after PTGBD in this study. Based on logistic regression, the severity of inflammation was the most significant factor in patient survival. It should be noted that Sanaiha et al*.* found significantly lower mortality in grade III inflammation after early LC than in percutaneous cholecystostomy based on a retrospective cohort of 358,624 patients [25]. This further elucidates the role of PTGBD as well as acute or early LC in the complex treatment of grade III AC.

In addition to mortality, the success of PTGBD is demonstrated by the need for emergency CCY, among other treatment options. In a systematic review by Winbladh et al*.*, the frequency of emergency CCY after percutaneous cholecystostomy was found to be between 2% and 20% [8]. In our study, emergency CCY was performed in 10% of the cases. These surgeries may become necessary when the disease progresses despite PTGBD and antibiotics or general supportive treatment. In the case noted above, extremely difficult surgeries can be expected with a high rate of primary open cholecystectomies (31.25%) and CR (18.18%), based on our study. The purpose of timely emergency CCY surgeries is to avoid complications, sepsis and septic shock as much as possible. We have to consider the fact that there is clinical progression after PTGBD in some cases and thus emergency CCY surgery should be performed (9.87%), which is a critical situation with high overall mortality (14.29%). In view of these results, the choice of emergency CCY or PTGBD should be considered in AC with grade III inflammation.

Several studies recommend percutaneous cholecystostomy in AAC [26, 27]. We should highlight that almost 56% mortality was observed after PTGBD among patients with AAC in our study. Due to the low number of cases, we have to interpret these results cautiously. However, a similar tendency was reported by Winbladh and his colleagues. In diagnostically uncertain cholecystitis, mortality was significantly higher than in AC with a clear origin; where the rate of gallstones was lower, mortality was expected to be higher, even up to 40–60% [8]. The previous observation was supported by a population-based analysis by Schlottmann et al*.* [28], in which a significantly worse postoperative outcome was observed in AAC than in patients with ACC after 7,516 cholecystostomy tube placement interventions.

Focussing on the proportion of elective cholecystectomies, it appears that cholecystectomy (CCY) was not performed after PTGBD in almost half of the cases (46.94%). Harai and his colleagues reported a similar trend: no CCY surgery was performed in almost 40% of cases after PTGBD [29]. Moreover, in a systematic review study of 1,925 patients by Winbladh and his colleagues, the proportion of patients who did not undergo surgery after percutaneous cholecystostomy was 62% [8]. PTGBD can be considered as a definitive final therapy [30]. This is also confirmed by our results, which showed that elective LCs generally occurred in younger patients (68.35 ± 11.34 years) compared to patients without further elective CCY (73.39 ± 15.39 years). Clarification of the causes of non-occurring CCY requires further investigation.

Emergency or urgent surgeries were needed in clinical progression and when the patient did not respond well to PTGBD intervention, especially in older patients with poor general condition (with high CCI) diagnosed with severe AC. Based on logistic regression analyses, the level of gallbladder inflammation has been significantly associated with the need for urgent surgery after PTGBD.

In the CRs of surgeries performed with different timings (elective or emergency), there was essentially no difference (17.46% *vs.* 18.18%). A similar result was obtained by Ni and colleagues [31], who found a conversion rate of 19.2% in patients who had previously undergone PTGBD. On average, the CR is around 4% during elective LCs [32, 33], but the CR of acute LC was around 9–10%. The remarkably high CR of elective LCs after PTGBD may be explained by the fact that these delayed LC surgeries were performed in older patients (68.35 ± 11.34 years), where, in addition to age, gallbladder wall thickening and adhesions from previous inflammation may further increase the chance of conversion. In these cases, it can be very difficult to accurately identify the structures of the Calot triangle that may lead to biliary tract injury. The ratio of major, Strasberg type D bile duct injury may be as high as 9.5% [34, 35]. In our study, the BDI ratio during CCY after PTGBD was 1.17%, which is roughly the same as the results reported by Altieri and her colleagues in 2019. According to Altieri, elective CCYs occurred in approximately 30% of patients after 9,738 PTGBDs, with a BDI rate of approximately 1.6% [36].

Following PTGBD, a relatively high portion (approximately 15% of patients) required ERCP. According to the literature, the need for ERCP can reach as high as 40% [37, 38]. In our study, ERCP was most often indicated due to the non-decreasing amount of bile excreted through the inserted drain. In most of the cases, the result of ERCP was biliary obstruction BO, including choledocholithiasis. We hypothesize that common bile duct stone (CBDS) played an important role in the development of AC in most patients, as AC more easily develops by increasing biliary pressure and interfering with gallbladder emptying. Based on our study, CBDS caused a problem indicating ERCP in approximately 44% of patients who underwent PTGBD. The importance of CBGS has been highlighted in several reports, including Kuan and her colleagues, who reported a 7–27% incidence of CBDS following percutaneous cholecystostomy [37, 38]. In mild or complete biliary obstruction, bile is emptied through a drain inserted into the gallbladder due to increased biliary pressure, which in many cases prevents the drain from being removed. Another cause of heavy bile excretion may be minor bile duct injury during a PTGBD procedure; however, no such case was observed in our study. In the light of this, it is recommended that ERCP be performed in cases where biliary excretion does not decrease after PTGBD.

## Conclusion

PTGBD is an easy-to-perform intervention in the treatment of acute cholecystitis with good TSR, with clinical and surgical success influenced by several factors, such as the patient’s age and the level of gallbladder inflammation. It is used as a definitive therapy in a significant proportion of patients, while it serves as bridging for other patients before subsequent elective CCY. The level of inflammation plays a crucial role in the course of AC, whereas in severe, grade III inflammation, we have to consider high clinical progression, a high proportion of emergency CCYs and high mortality after PTGBD.

### Limitations of the study

Limitations include retrospective nature of the study, with relative restrictions in patient selection and inclusion. We were unable to identify patients who had died outside our institutions; therefore, we could not determine the exact number and causes of the 30-day and overall mortality. After PTGBD, we were unable to identify the direct causes of the absence of elective CCY, so we can only infer them.
